# Test–Retest Reliability of a Computerized Hand–Eye Coordination Task

**DOI:** 10.3390/jemr18050054

**Published:** 2025-10-14

**Authors:** Antonio Ríder-Vázquez, Estanislao Gutiérrez-Sánchez, Clara Martinez-Perez, María Carmen Sánchez-González

**Affiliations:** 1 Department of Physics of Condensed Matter, Optics Area, University of Seville, Reina Mercedes S/N, 41012 Seville, Spain; antonioriderv@gmail.com (A.R.-V.); msanchez77@us.es (M.C.S.-G.); 2 Department of Surgery, Ophthalmology Area, University of Seville, Doctor Fedriani S/N, 41009 Seville, Spain; egutierrez1@us.es; 3 Instituto Superior de Educação e Ciências de Lisboa (ISEC Lisboa), Alameda das Linhas de Torres, 179, 1750-142 Lisboa, Portugal

**Keywords:** hand–eye coordination, visual–motor performance, test–retest reliability, response time, intra- and inter-examiner reproducibility, digital assessment, COI-SV^®^

## Abstract

**Background:** Hand–eye coordination is essential for daily functioning and sports performance, but standardized digital protocols for its reliable assessment are limited. This study aimed to evaluate the intra-examiner repeatability and inter-examiner reproducibility of a computerized protocol (COI-SV^®^) for assessing hand–eye coordination in healthy adults, as well as the influence of age and sex. **Methods:** Seventy-eight adults completed four sessions of a computerized visual–motor task requiring rapid and accurate responses to randomly presented targets. Accuracy and response times were analyzed using repeated-measures and reliability analyses. **Results:** Accuracy showed a small session effect and minor examiner differences on the first day, whereas response times were consistent across sessions. Men generally responded faster than women, and response times increased slightly with age. Overall, reliability indices indicated moderate-to-good repeatability and reproducibility for both accuracy and response time measures. **Conclusions:** The COI-SV^®^ protocol provides a robust, objective, and reproducible measurement of hand–eye coordination, supporting its use in clinical, sports, and research settings.

## 1. Introduction

Hand–eye coordination is a fundamental capacity of the central nervous system that enables the use of visual information to guide and control manual movements such as reaching, writing, or catching objects [1,2,3]. This skill requires efficient integration of the visual system, motor pathways, and upper limbs, functioning as a sequential process from the visual detection of the target to muscle activation [1,3,4].

Its development begins in childhood and improves throughout life with practice and learning [5,6,7]. Activities like building games or object manipulation stimulate perception–action coupling, promoting maturation of cortical and subcortical circuits involved in movement planning and execution [5,8,9]. Neurologically, it relies on regions such as the cerebellum, posterior parietal cortex, and basal ganglia, as well as connections between visual and motor areas [8,9].

Functionally, gaze–hand coordination follows an ordered sequence: visual localization, attentional focus, perceptual identification, motor planning, and finally muscle activation [1,2,4]. Although eye movements are completed faster than manual ones, both actions are tightly coupled [4,10,11], adapting to spatial and temporal demands, as shown in pointing tasks using the index of difficulty paradigm [11,12,13]. When precision requirements increase, each reaching movement is preceded by an eye saccade, whereas lower demands allow intermittent control [12,13,14,15].

Interest in this capacity extends beyond clinical and neurological contexts. In sports such as volleyball, tennis, or badminton, it is a determinant of performance, with training improving visual reaction speed and inducing structural brain adaptations [16,17,18,19,20]. Beyond sports, hand–eye coordination is essential in daily activities—from writing or driving to digital interaction—underpinning functionality, independence, and learning [2,3,21]. Performance is also shaped by age and sex: coordination patterns mature around age 10, and some studies report task-dependent sex differences [6,7,8,9,22].

In clinical and research practice, a key challenge is the availability of standardized tools that reliably measure hand–eye coordination. While tests exist for isolated components such as precision or reaction time, protocols combining both are less common and require validation. Assessing inter- and intra-examiner reliability and session-to-session stability is essential for their diagnostic and training use [23]. The integration of eye tracking and digital recordings offers new opportunities, but robust validation remains needed [24,25,26,27].

The objective of this study was therefore to analyze the intra-examiner repeatability and inter-examiner reproducibility of a computerized method for hand–eye coordination, and to examine the influence of age and sex.

## 2. Materials and Methods

### 2.1. Study Design

A prospective study was conducted at the “Centro de Optometría Internacional” (COI) facilities in Madrid, Spain, between May and June 2023. A total of 78 individuals (42 males and 36 females) participated in the research. All participants gave their informed consent in accordance with ethical guidelines. The study adhered to the principles of the Declaration of Helsinki (1964) and received approval from the Research Ethics Committee of the University of Seville.

### 2.2. Participant Recruitment

Sample size estimation was carried out using the Granmo calculator (version 7.12; Institut Municipal d’Investigació Médica, Barcelona, Spain) [28]. According to the study parameters, a minimum of 46 participants was needed to achieve 80% statistical power, with a significance level (alpha) of 0.05 and a beta risk of 0.2 for a two-sided test.

Eligible participants were adults aged 18 to 65 years, with binocular and monocular distance visual acuity (VA) of 20/25 or better while wearing their distance correction. Exclusion criteria included any pathological condition causing visual field limitations, impairments in reaction time or eye movement, chronic medication use that could affect reaction time, intake of acute medication within 24 h prior to testing, and consumption of alcohol, drugs, or other substances potentially impacting test performance within 24 h before the assessment.

Participants were recruited through public advertisements and word of mouth at the COI facilities. Interested individuals contacted the research team and were screened against inclusion and exclusion criteria using a short questionnaire and an initial visual examination. A total of 78 respondents were enrolled (42 men, 36 women; mean age 32.5 ± 11.7 years).

### 2.3. Hand–Eye Coordination

This measurement was based on the Acuvision 1000^®^ [29], a tool created to improve hand–eye coordination in athletes. During the test, 120 red dots appeared randomly and evenly across the four sections of the visual field. Participants were required to touch these dots as quickly and accurately as possible. The recorded data included the number of correct and missed touches, along with the average reaction time overall and for each visual quadrant.

Hand–eye coordination was measured using the COI Sport Vision^®^ (COI-SV^®^) digital software. Developed by “Centro de Optometría Internacional” (Madrid, Spain), this software included diagnostic and treatment tests specifically designed for sports vision [30]. The software had an excellent reliability (α = 0.93) and had been used in other similar studies [31,32]. To reduce examiner-related biases, the procedure was standardized as follows:

1. The participant was seated approximately 60–70 cm from the screen.
2. The examiner read aloud the standardized instructions: *“Red dots will appear randomly anywhere on the screen. You must try to touch the red dot as quickly and accurately as possible. You may use whichever hand you prefer.”*
3. The participant confirmed understanding of the instructions.
4. The test was initiated with the command: *“Let’s begin.”*

### 2.4. Test and Re-Test Methodology

**Figure 1:**
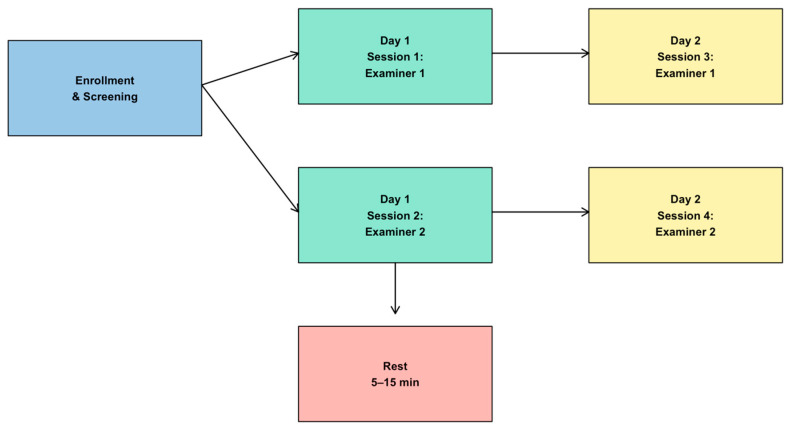
Experimental protocol. Schematic representation of participant flow across the four test sessions: Day 1 (Examiner 1 and Examiner 2) and Day 2 (Examiner 1 and Examiner 2), with a two-week interval between days and 5–15 min rest periods between sessions.

To evaluate the repeatability of the measurement method, a protocol was established in which each participant performed the tests twice across two separate days, resulting in a total of four trials under the same conditions [30,33,34,35]. In this study, Session 1 corresponds to Examiner 1 on Day 1, Session 2 to Examiner 2 on Day 1, Session 3 to Examiner 1 on Day 2, and Session 4 to Examiner 2 on Day 2. This designation is used consistently throughout the manuscript and in the figures. On the initial day, participants underwent preliminary assessments and completed a short questionnaire to verify compliance with inclusion and exclusion criteria. Each participant was evaluated by two different examiners; the order in which examiners conducted the tests was randomized on the first day and reversed for the second day. Examiners did not have access to each other’s results or to their own previous assessments. Both examiners were optometrists with comparable clinical and research experience in visuomotor testing. A third examiner was responsible for securely managing and storing the data. Moreover, participants were kept unaware of their earlier test results. Tests were spaced with rest periods ranging from 5 to 15 min, and approximately two weeks separated the first and second testing sessions. The overall structure of the experimental protocol is summarized in Figure 1.

### 2.5. Statistical Analysis

Statistical analyses were performed using R (version 4.4.2). An initial description of the sample was conducted, including measures of central tendency and dispersion for age, as well as distribution by sex.

To assess intra- and inter-examiner reproducibility of the hand–eye coordination task, data on correct responses and mean reaction times from four sessions (two per examiner on different days) were analyzed. The variables were converted to long format to facilitate the analyses.

Differences between sessions and examiners were evaluated using repeated-measures analysis of variance (ANOVA), including fixed effects for examiner, day, and their interaction, and a random effect for participant. Accuracy scores were treated as continuous variables given their approximately normal distribution. A sensitivity analysis using a Poisson generalized linear mixed-effects model yielded consistent results, confirming the robustness of the findings.

Test–retest reliability was estimated using intraclass correlation coefficients (ICCs) with a two-way random-effects, absolute-agreement, single-measures model (ICC [2,1]). To explore systematic bias and variability between measurements, Bland–Altman plots were computed with 95% limits of agreement (LoA), defined as the mean of the differences ± 1.96 times the standard deviation of the differences.

Paired *t*-tests were conducted to compare performance between examiners in the same session (inter-examiner) and between sessions of the same examiner (intra-examiner). Correlations between age and performance variables (accuracy and time) were calculated using Spearman’s coefficient. In addition, sex differences were evaluated using independent *t*-tests. The simultaneous influence of age and sex on reaction time was examined using an ANCOVA model. Finally, the relationship between accuracy and speed (the classic accuracy–latency trade-off) was evaluated through bivariate correlations by session. A *p*-value < 0.05 was considered statistically significant.

## 3. Results

### 3.1. Participants

After excluding two participants (one ineligible and one who did not complete the test), a total of 78 participants were included in the final analysis, comprising 42 men and 36 women. The mean age was 32.50 years (SD = 11.70), the median age was 27 years, and the interquartile range (IQR) was 13.80 years. Participant ages ranged from 19 to 64 years.

**Figure 2:**
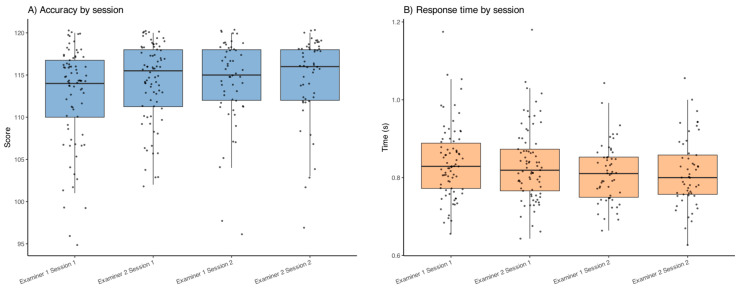
Mean accuracy scores (**A**) and response times (**B**) per session.

Figure 2A shows the mean scores obtained in each session. The repeated measures ANOVA revealed a statistically significant difference in mean scores across sessions (F(3,183) = 11.03, *p* = 0.002), suggesting a small but significant session effect on accuracy. A sensitivity analysis using a Poisson generalized linear mixed-effects model confirmed this session effect (*p* < 0.05), indicating that the result was not dependent on the statistical approach. Additionally, a paired *t*-test comparing Examiner 1 Session 1 and Examiner 2 Session 1 showed a significant difference (t = −2.51, *p* = 0.014), with lower scores observed in Examiner 1 Session 1.

Regarding response time, Figure 2B presents the mean times per session. The repeated measures ANOVA did not show significant differences in mean response time across sessions (F(3,181) = 2.05, *p* = 0.158), indicating similar temporal performance regardless of session or examiner. The visualization confirms a consistent temporal pattern across all sessions, with low variability among participants.

### 3.2. Influence of Age and Sex

**Figure 3:**
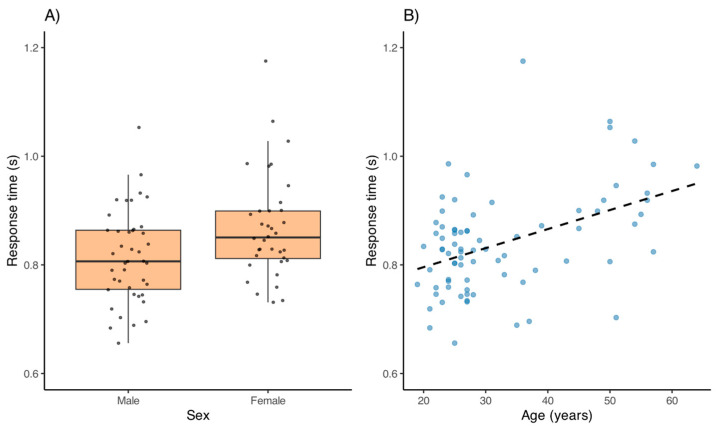
(**A**) Response time by sex in Session 1, showing lower mean times in men. (**B**) Scatter plot illustrating the positive association between age and response time in Session 1.

No significant differences in accuracy scores were found between men and women across sessions, nor were there significant associations between age and accuracy (all *p* > 0.05). In contrast, response time showed a significant sex difference in Session 1 (*p* = 0.011), with men being faster (0.81 ± 0.08 s) than women (0.87 ± 0.10 s), as illustrated in Figure 3A. In other sessions, no significant sex differences were detected (all *p* > 0.05), although a trend toward faster times in men was observed. Age was positively correlated with response time in all sessions (Session 1: r = 0.35, *p* = 0.002; Session 2: r = 0.27, *p* = 0.019; Session 3: r = 0.31, *p* = 0.024; Session 4: r = 0.32, *p* = 0.018), indicating that older participants tended to respond more slowly (Figure 3B). An analysis of covariance (ANCOVA) performed for Session 1 confirmed that both sex (*p* = 0.006) and age (*p* < 0.001) had significant effects on response time, suggesting that sex differences persisted even after adjusting for age.

Furthermore, correlation analyses revealed significant positive associations between accuracy scores and response times in all sessions (Session 1: r = 0.47, *p* < 0.001; Session 2: r = 0.56, *p* < 0.001; Session 3: r = 0.44, *p* < 0.001; Session 4: r = 0.54, *p* < 0.001), confirming the classic speed–accuracy trade-off, where higher precision was associated with longer response times. This pattern indicates that participants prioritized accuracy at the cost of speed, consistent with previous visuomotor research findings.

### 3.3. Reproducibility and Session Effects

A linear mixed-effects model was applied to assess the effects of examiner, day (Day 1 vs. Day 2), and their interaction on both accuracy and response time, using participant as a random effect. For accuracy scores, the model revealed significant main effects of examiner (F(1,183) = 7.55, *p* = 0.007) and day (F(1,183) = 7.26, *p* = 0.008). The interaction between examiner and session was not significant (F(1,183) = 1.17, *p* = 0.281), indicating independent effects.

For response time, the effect of examiner was not significant (F(1,181) = 0.26, *p* = 0.613), nor was the interaction term (F(1,181) = 0.32, *p* = 0.572). However, a significant day effect was observed (F(1,181) = 4.71, *p* = 0.031), indicating slightly faster or slower response times depending on the testing day.

### 3.4. Reproducibility and Reliability Analysis

**Figure 4:**
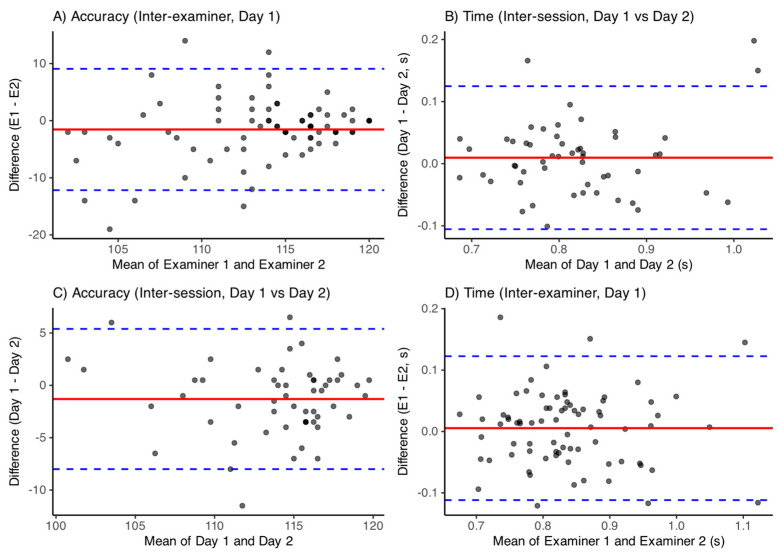
Bland-Altman plots illustrating agreement for accuracy and response time measures. (**A**) Inter-examiner agreement for accuracy scores (Day 1). (**B**) Inter-session agreement for response time (Day 1 vs. Day 2). (**C**) Inter-session agreement for accuracy (Day 1 vs. Day 2); (**D**) Inter-examiner agreement for response time (Day 1). The solid horizontal line represents the mean bias, and the dotted lines indicate the 95% limits of agreement.

Intra-session repeatability was assessed by comparing accuracy scores and response times between Day 1 and Day 2 (mean across examiners). Inter-examiner reproducibility was evaluated separately for each day, and intra-examiner reproducibility was examined for each examiner. A summary of mean biases, 95% limits of agreement, and ICC values is presented in Table 1. Overall, both accuracy and response times showed small biases, narrow limits of agreement, and moderate-to-good ICCs, indicating robust reproducibility across sessions and examiners. Corresponding Bland–Altman plots (Figure 4A–D) illustrate these findings.

## 4. Discussion

This study provides evidence for the repeatability and reproducibility of a computerized protocol (COI-SV^®^) designed to assess hand–eye coordination in healthy adults. Both accuracy and response time demonstrated acceptable stability across sessions and examiners, supporting the utility of this protocol in clinical, sports, and research contexts where objective monitoring of visuomotor performance is required.

A small session effect on accuracy was detected, with slightly higher scores in subsequent sessions. Similar findings have been reported in studies of reaching or precision tasks, where repeated exposure produces limited improvements in motor accuracy [16,36,37]. In our cohort of untrained adults, however, these differences were modest and are more likely attributable to natural variability of the test rather than true learning effects. In fact, recent reliability studies of computerized visuomotor tests have reported ICC values in the range of 0.80–0.92 for both upper- and lower-extremity visuomotor reaction time tasks [38] and 0.86–0.94 for VR-based coordination and reaction time tests [39]. Our intra-session ICC of 0.70 and inter-examiner ICCs of 0.51–0.71 therefore fall within the same order of magnitude, supporting that the stability observed in this study is consistent with the expected performance of similar instruments. Importantly, the significant examiner difference observed in the first session aligns with previous reports attributing discrepancies to subtle variations in test instructions or initial participant familiarization [23], underlining the importance of strict standardization.

Response time measures showed greater robustness than accuracy. No systematic differences were observed across sessions or between examiners, in agreement with previous findings that temporal variables in computerized visuomotor tasks are less affected by contextual variability or fatigue [23,40]. This temporal stability is particularly valuable in longitudinal monitoring, where reliable detection of change depends on minimizing measurement error. At the same time, individual factors shaped temporal performance: men were faster than women in the first session, a difference that diminished with task repetition, and older participants consistently required longer times. These findings reflect well-documented patterns in the literature [36,40,41], where sex differences are often more pronounced during initial exposure and age-related slowing is a robust population trend.

A consistent positive correlation was observed between accuracy and response time across all sessions, confirming the classic speed–accuracy trade-off described by Fitts [42]. This relationship indicates that participants who aimed for greater precision did so at the cost of longer latencies, a phenomenon widely reported in both experimental and applied contexts [23,40]. For practical applications, this reinforces the need to interpret both accuracy and speed jointly, rather than in isolation, when evaluating visuomotor ability.

In terms of reproducibility, the Bland–Altman analysis confirmed small biases and acceptable limits of agreement for both intra- and inter-examiner comparisons, indicating that observed variations are unlikely to reflect true performance changes. These findings align with previous computerized reliability studies in sports and clinical settings [16,23,43,44], and support the use of the COI-SV^®^ protocol as a stable monitoring tool. Compared to other available tools, such as Acuvision, Leap Motion, or EyeTribe, the COI-SV^®^ protocol offers a number of practical advantages. It can be implemented on standard computer hardware without the need for specialized VR or haptic devices, making it more accessible and cost-efficient. At the same time, it provides standardized, reproducible measurements that are less dependent on examiner influence or contextual variability. Unlike fully immersive VR approaches, COI-SV^®^ maintains ecological validity by replicating digital interaction contexts common in everyday life, while still allowing for precise quantification of visuomotor performance. Thus, rather than replacing immersive or haptic systems, COI-SV^®^ complements them by offering a fast, scalable, and objective tool suitable for baseline screening, telehealth applications, and longitudinal monitoring.

Several limitations should be acknowledged. The study population consisted exclusively of healthy adults, limiting the generalizability of the findings to children, older adults, or individuals with neurological or visual disorders. The protocol did not include more complex task demands such as bimanual coordination, multitasking, or fatigue conditions, which may influence performance in applied settings. Another limitation concerns ecological validity: while the COI-SV^®^ task provides standardized and reproducible measurements, tapping digital red dots may not fully reflect the visuomotor complexity of real-world contexts such as sports performance or clinical rehabilitation, where additional perceptual, motor, and decision-making demands are present. These considerations highlight the need for future studies to extend validation to different populations and more complex testing scenarios.

Future research should also examine the sensitivity of the COI-SV^®^ protocol in detecting changes after visuomotor training, clinical rehabilitation, or neurological interventions. Validation in clinical cohorts where hand–eye coordination is critical, such as stroke survivors, individuals with Parkinson’s disease, or children with ADHD, would provide further evidence of its clinical applicability. Integrating the platform with emerging technologies such as real-time eye tracking, haptic feedback, or immersive VR could enhance ecological validity and provide richer insights into visuomotor control. Longitudinal applications are also promising, since the COI-SV^®^ could be used to track recovery trajectories in rehabilitation or to monitor progressive improvements following sports or therapeutic training. Such studies will be important to distinguish true performance changes from intra-individual variability and measurement error.

From a practical perspective, the COI-SV^®^ protocol represents a reliable and accessible tool for the objective assessment of hand–eye coordination. Its potential applications extend from baseline evaluations and follow-up in rehabilitation programs to functional assessments in sports performance, optometry, and neurology. The ability to obtain precise and comparable measurements across sessions and examiners facilitates the detection of clinically meaningful changes, supports individualized interventions, and allows integration into telemedicine and digital health platforms.

## 5. Conclusions

In conclusion, this study confirms that the computerized assessment of hand–eye coordination using the COI-SV^®^ protocol is a reliable, objective, and reproducible tool for use in healthy adults. Both accuracy and response time demonstrated remarkable stability across sessions and examiners, supporting the methodological robustness of the test and its potential utility for monitoring purposes in clinical, sports, and research settings.

The absence of significant sex- or age-related differences in accuracy, along with the identification of the classic speed–accuracy trade-off, reinforces the value of this protocol for comprehensive visuomotor function assessment. While the learning or familiarization effects were minimal, the study highlights the importance of standardized administration to maximize measurement reliability.

Although the results should be interpreted in light of the sample characteristics and evaluation context, the computerized test offers a solid foundation for future clinical applications and research involving diverse populations, functional settings, and emerging assessment technologies.

## Figures and Tables

**Table 1:** Summary of reproducibility analyses for accuracy and response time.

Comparison	Measure	Bias (Mean Difference)	95% LoA	ICC (95% CI)
Intra-session (Day 1 vs. Day 2)	Accuracy	+1.31 points	−5.39 to 8.00	0.70 (0.52–0.82)
	Response time	−0.01 s	−0.13 to 0.11	
Inter-examiner (Day 1)	Accuracy	+1.33 points	−8.54 to 11.20	0.51 (0.28–0.68)
	Response time	−0.01 s	−0.12 to 0.11	
Inter-examiner (Day 2)	Accuracy	+0.61 points	−6.98 to 8.20	0.71 (0.55–0.82)
	Response time	−0.001 s	−0.09 to 0.08	
Intra-examiner (Examiner 1)	Accuracy	+1.67 points	−8.49 to 11.82	—
Intra-examiner (Examiner 2)	Accuracy	+0.94 points	−8.20 to 10.09	—

## Data Availability

The raw data supporting the conclusions of this article will be made available by the authors on request.
